# Vision-Based Facial Analysis for Screening and Symptom Estimation of Mental Health Conditions: A Critical Narrative Review

**DOI:** 10.3390/s26206437

**Published:** 2026-10-12

**Authors:** Yaohua Guan, Guangqiang Yin

**Affiliations:** 1 School of Information and Software Engineering, University of Electronic Science and Technology of China, Chengdu 611731, China; 202112281014@std.uestc.edu.cn; 2 Shenzhen Institute for Advanced Study, University of Electronic Science and Technology of China, Shenzhen 518000, China

**Keywords:** mental health screening, symptom estimation, facial behaviour, remote photoplethysmography, camera sensing, validation, multimodal learning

## Abstract

Facial video provides behavioural measurements and camera-derived physiological signals that may complement mental health assessment. Their clinical interpretation, however, depends on the acquisition conditions, reference labels, population, and evaluation protocol. This critical narrative review examines facial analysis for mental health screening and symptom estimation, distinguishing depression, anxiety symptoms, experimentally elicited stress, and other condition-specific assessment tasks. We distinguish questionnaire-defined screening labels, symptom severity, experimentally elicited affect or stress, and clinician-established diagnoses. We review image acquisition and preprocessing, facial action units and movement, remote photoplethysmography (rPPG), temporal architectures, and multimodal fusion. Dataset resources are separated by evidential purpose, and representative quantitative results are accompanied by their targets, modalities, and validation conditions rather than presented as a cross-study ranking. The synthesis identifies three recurring limitations: few independent participants despite many video frames, limited validation across populations and recording domains, and a gap between benchmark prediction and clinical utility. Accurate average pulse rate estimation does not establish reliable beat-to-beat variability or disorder-specific validity. We therefore emphasise participant-level evaluation, probability calibration, prevalence-dependent predictive values, acquisition quality, consent, and prospective clinical assessment. Foundation models and longitudinal monitoring remain research directions whose incremental value must be established against appropriate baselines. Current evidence supports investigation of complementary screening and decision support tools, rather than autonomous diagnosis or a demonstrated ability to predict disease onset.

## 1. Introduction

### 1.1. Scope and Motivation

Facial video is a potential source of complementary information in mental health assessment because it records movement, expression, gaze, and skin-reflectance changes during an interaction. It can be acquired alongside an interview without attaching a contact sensor. These advantages motivate investigation of complementary measurements. Section 2 defines their target and validation requirements.

The review covers facial analysis in depression, anxiety and stress research, alongside selected condition-specific applications. The most developed quantitative examples in the available material concern depression-related labels; this imbalance should not be read as equivalent evidence across conditions. Anxiety symptom estimation, acute stress recognition and assessment within a diagnosed cohort are therefore discussed as separate tasks. General affect recognition and cardiovascular measurement are treated as enabling technologies, not as equivalent mental health outcomes. Most studies estimate concurrent symptoms or labels. Prediction before disease onset is a separate longitudinal task and is not implied by the title.

Clinical assessment combines information about symptom duration, impairment, medical conditions, medication, context, and differential diagnoses. Facial measurements cover only part of this process. The relevant engineering question is therefore whether a defined signal adds reliable information to an existing assessment workflow for a specified population and intended use. More complex models, larger image counts, or better within-corpus scores alone do not answer that question.

### 1.2. Relationship to Previous Reviews

**Figure 1:**
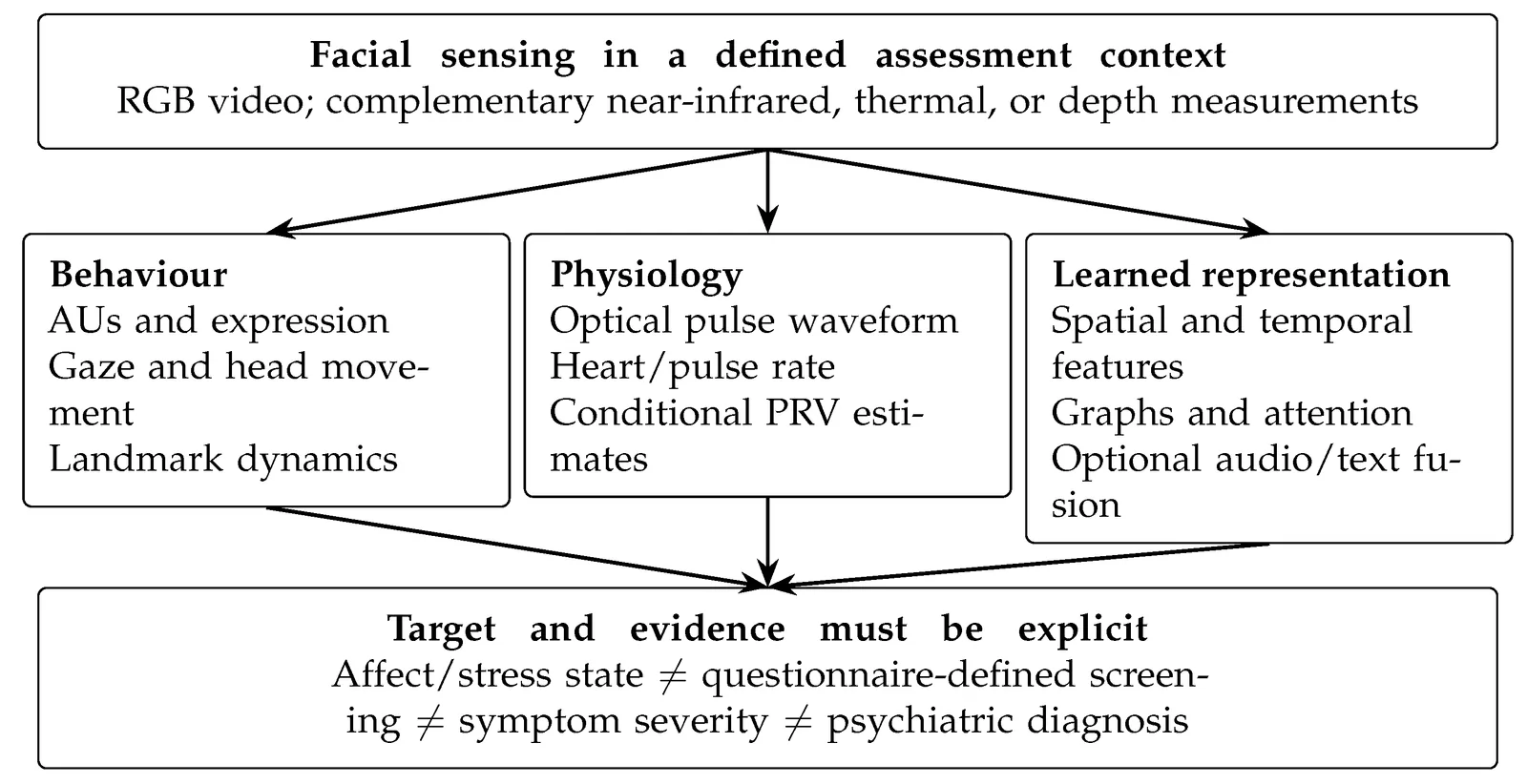
Scope of facial sensing. The three representation routes can overlap; a learned model can estimate AUs or pulse signals. None determines the clinical meaning of its target without an appropriate reference standard and validation. PRV denotes pulse rate variability.

Prior work includes the visual depression review by Pampouchidou et al. [1] and the audiovisual reviews by He et al. and Han et al. [2,3]. Sakib et al. review camera-derived physiological measurement [4]; the present contribution is not the first facially focused survey. Anxiety also has a dedicated machine-learning review covering multiple signal types, which provides a broader context for the facial component considered here [5]. Stress detection and passive sensing reviews additionally place facial input within broader multimodal systems [6,7]. Table 1 locates this review relative to these contributions. Our emphasis is the connection between acquisition constraints, the clinical meaning of labels, and the conditions under which quantitative results can be interpreted. rPPG is included as a complementary sensing route, with separate consideration of pulse measurement validity and mental health validity. Figure 1 summarises this scope at its first discussion.

### 1.3. Review Approach and Evidence Selection

This article is a critical narrative review of facial sensing for mental health screening and symptom estimation, rather than a systematic review or meta-analysis. Literature collection drew on IEEE Xplore, ACM Digital Library, SpringerLink, ScienceDirect, PubMed, and Google Scholar, using combinations of facial expression, depression, anxiety, stress, mental health screening, rPPG, facial action units, and deep learning. The main publication emphasis is 2019–2026, particularly 2022–2026; seminal earlier studies are retained to explain clinical measures and enabling technologies. The targeted literature update for this revision was completed on 30 September 2026. Backward citation checking was used selectively to follow relevant references in selected reviews and primary papers, alongside searches for the reviewer-nominated works. No exhaustive forward-citation search was undertaken. This date identifies the last targeted update, not a claim that every database was searched exhaustively on that day.

Selection is guided by relevance to facial visual or face-derived physiological measurements, clarity of the target and reference standard, and the contribution to understanding methods, datasets, evaluation or clinical interpretation. Empirical studies are used for quantitative comparison when a reported value can be linked to an identifiable task and evaluation setting. Generic affect and pulse-estimation studies are included as enabling evidence, not psychiatric validation. Work using only audio or text is discussed only when it clarifies the role of video acquisition or the need to establish the incremental value of facial information. Publications with insufficiently characterised performance are discussed qualitatively or with explicit limits on comparability. This purposive selection does not support an exhaustive study count or a PRISMA selection flow claim; the bibliography includes background and methodological references as well as empirical studies.

For synthesis, sources are separated by evidential role. Empirical mental health studies link facial visual or face-derived physiological input to a specified screening label, symptom measure, stress condition, or clinical assessment. Dataset descriptions establish population, acquisition, reference labels, and available input. Foundational vision, affect recognition and rPPG papers explain enabling components without being counted as psychiatric validation. Clinical measurement and official regulatory sources support interpretation and intended-use discussion. A study collected through a video interface does not qualify as evidence for facial analysis if its model uses only audio or text.

The evidence tables record the dataset, target and reference standard, input modality, evaluation partition, metric, and reported value. Population denominators are given in the dataset descriptions. A value with a verified source but incomplete protocol information is retained with that limitation, rather than assigned an assumed participant split. Results reproduced in another paper are distinguished from direct extraction. Comparisons are qualified by target, scale, population, split and acquisition conditions; no pooled effect or universal architecture ranking is inferred. Because selection is purposive and full reporting is uneven, this synthesis may omit relevant studies and does not estimate pooled performance or the frequency of particular findings across the entire literature.

The remainder of the review follows the measurement chain. Section 2 defines tasks and labels; Section 3 examines acquisition, preprocessing, and representations; Section 4 compares model families; Section 5 groups datasets and interprets quantitative evidence. Section 6, Section 7 and Section 8 address intended uses, deployment constraints, and research priorities.

## 2. Clinical Tasks, Reference Standards, and Interpretation

### 2.1. Screening, Symptom Estimation, State Recognition, and Diagnosis

Four targets must be distinguished. *Screening* identifies individuals who may benefit from further assessment; *symptom estimation* predicts the level of a specified symptom scale; *affect or stress recognition* estimates a transient or experimentally elicited state; and *clinical diagnosis* establishes a disorder through an appropriate clinical assessment. These targets differ in timescale, reference standard and clinical consequence. Predicting a questionnaire-defined group is therefore described here as screening-label prediction, rather than diagnosis. Recognising a brief stress response or an expression category does not by itself establish an anxiety disorder or depression. Likewise, an interview recorded after diagnosis does not provide evidence of detection before clinical onset. A claim of early prediction requires longitudinal follow-up, a clearly defined outcome, and a prespecified prediction horizon.

### 2.2. What the Reference Standard Measures

The PHQ-8 has a score range of 0–24, whereas the PHQ-9 ranges from 0 to 27 [9,10]. A threshold such as PHQ-9 ≥10 defines a commonly used screening-positive group; it is not a universal diagnostic criterion. The GAD-7 is an anxiety symptom instrument [11], while the DASS-21 measures depression, anxiety, and stress dimensions. The DASS developers explicitly distinguish its dimensional scores from assignment to DSM or ICD diagnostic categories [12]. Clinician-rated symptom severity, such as the Hamilton Depression Rating Scale, is also distinct from the procedure used to establish diagnosis or cohort eligibility [13].

Consequently, dataset descriptions should distinguish questionnaire-defined labels, clinician-rated severity, structured diagnostic interviews, and formally diagnosed cohorts. They should report the instrument version, scale range, threshold, assessor and interval between recording and assessment. A clinically recruited dataset may still use self-reported severity as its machine-learning target; recruitment setting does not change the meaning of that target. Conversely, an affect-pretraining dataset can help learn facial representations without supplying mental health ground truth. Symptom-scale MAE should be interpreted on the original scale, and scores from different instruments should not be treated as interchangeable merely because both concern depression.

### 2.3. Facial Behaviour: Context-Dependent Associations

Facial measurements are potential complementary digital markers, with context-dependent associations rather than disorder-specific signatures. In a longitudinal clinical-interview study, Girard et al. reported reduced head-motion amplitude and velocity at higher depression severity. Manual coding identified reduced AU12 and AU15 and increased AU14; the automated AU12 difference did not reach significance [14]. The direction of the AU15 result illustrates why a fixed list of movements should not be presented as a universal depressive facial profile. The study also concerns behaviour within a particular interview and treatment context, which limits extrapolation to unconstrained recordings.

Gaze, head movement and expressivity can encode interpersonal behaviour, interview content, habitual movement, and recording artefacts as well as clinically relevant variation. Their interpretation therefore requires the elicitation context, measurement reliability, and participant characteristics. For anxiety and stress, the label must specify whether it represents a questionnaire score, a clinical assessment, a self-reported momentary state or an experimental condition. The target distinctions in Box 1 apply to these labels.

Box 1 **Principles for interpreting the evidence.**

1. **Match the claim to the reference.** Questionnaire positivity, symptom severity, induced stress and clinical diagnosis are separate targets; concurrent prediction is not prediction before onset.
2. **Match the split to the intended user.** New-user generalisation requires participant-disjoint data, including repeated sessions and derived clips. Frame-, session- and channel-disjoint splits support different claims.
3. **Separate validation layers.** Pulse accuracy, symptom prediction and improved clinical decisions require their own endpoints. Good average heart rate estimation alone does not validate pulse-interval variability.
4. **Interpret results within their setting.** Compare matched targets and protocols, report uncertainty and subgroup denominators, and assess added value in the intended population before deployment.

### 2.4. Physiological Measurement Is a Separate Validation Layer

For rPPG, the validation layers in Box 1 require a contact-reference assessment of physiological recovery, an evaluation against the intended symptom or stress target, and a study of clinical utility. Medication, physical activity, sleep, cardiovascular status, and acquisition conditions should be recorded as potential alternative explanations. These factors guide confounder analysis and choice of comparator rather than being assigned a psychiatric meaning from the pulse trace alone.

### 2.5. Anxiety, Acute Stress and Condition-Specific Tasks

The reference target matters even when the same facial feature is used. Giannakakis et al. studied AU-based acute stress recognition in an experiment with 58 participants, four phases and 11 stress/non-stress tasks, using feature selection and relevance propagation [15]. This supports a task-specific investigation of facial movements, rather than a universal anxiety disorder signature. Speech, task demands, and the way stress is induced need to be considered alongside the selected AUs. FACES adds questionnaire-linked anxiety and stress results in adolescents, summarised in Section 5.2 [16]. These sources offer complementary targets, but limited independent clinical validation and differing elicitation settings prevent treating their results as one evidence base for anxiety disorders.

Panickar and Gayathri propose a face-parsing framework for adolescent anxiety assessment [17]. Its reported experiments evaluate an emotion recognition component on CK+ and FER2013. We therefore include it as an enabling representation approach, rather than as a questionnaire-linked anxiety result.

Condition-specific assessment also extends beyond depression. Ceccarelli and Mahmoud evaluated temporal multimodal representations and adaptive late fusion on a bipolar disorder (BD) corpus for state classification [18]. Assessing state within a bipolar cohort is different from detecting bipolar disorder in an unselected population. Their reported development-set result is retained with that distinction in Section 5.2. Evidence for one condition or population does not establish validity for another; reference standards, treatment status, and intended use must be specified for each application.

## 3. Facial Sensing and Feature Extraction

**Figure 2:**
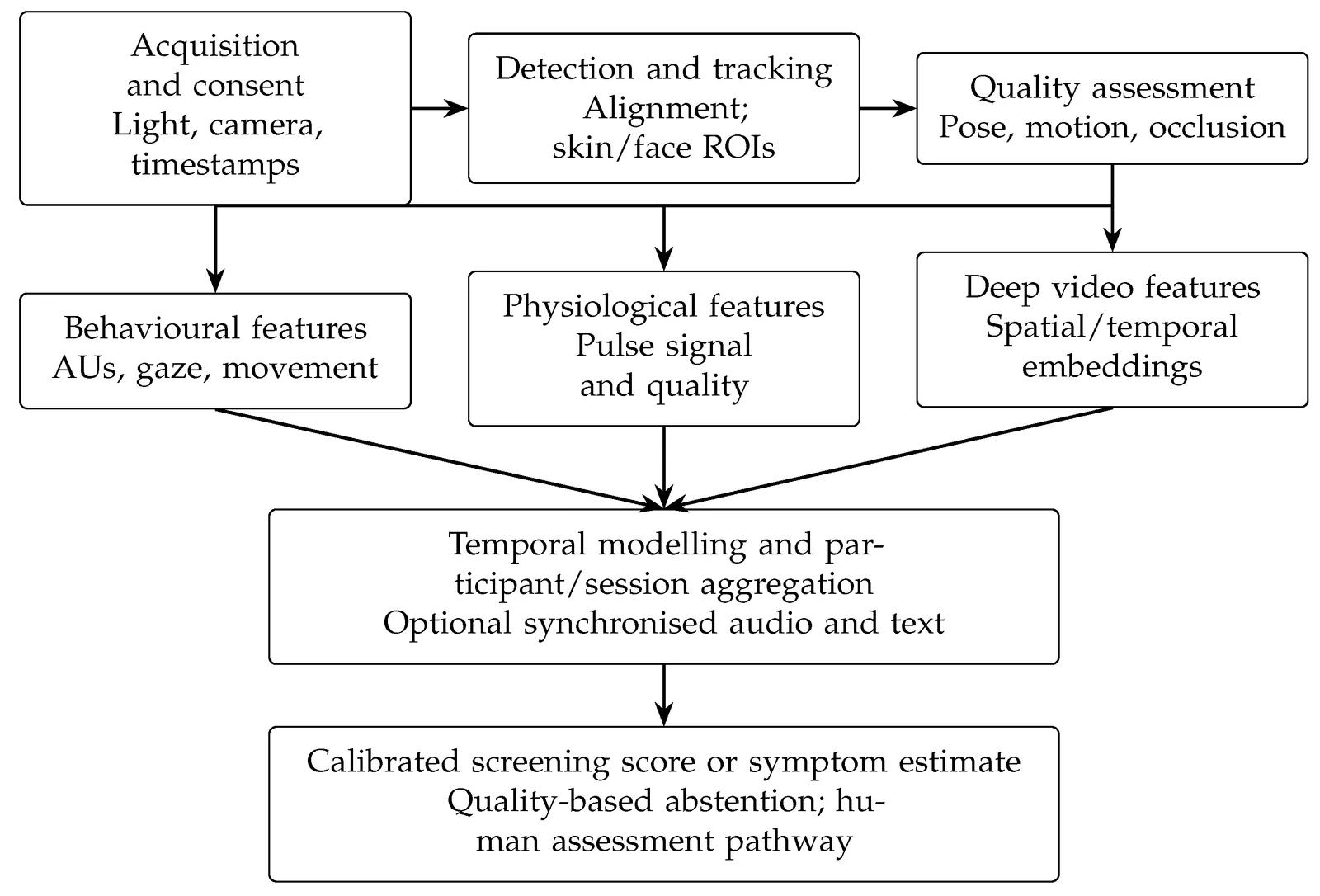
From acquisition to a defined output. Quality failures should be propagated to the final output and can require abstention. Aggregation and all learned preprocessing must be evaluated without participant leakage; a face crop is not an independent patient.

The acquisition, preprocessing, and representation stages form one measurement chain. Figure 2 shows the common stages and the behavioural, physiological, and learned-feature routes. Their quality requirements differ, and a failure in an early stage can propagate to an apparently precise final score.

### 3.1. Acquisition Modalities and Measurement Constraints

The camera is part of the measurement model, rather than a neutral source of images. Behavioural analysis uses spatial appearance and movement, whereas rPPG seeks much smaller changes in skin reflectance associated with pulsatile blood volume. Consequently, a video that is visually adequate for an interview may be unsuitable for pulse measurement. The optical model underlying POS explicitly distinguishes physiological colour variation from illumination and surface-reflection effects [19]. Acquisition reports should therefore specify camera model, spectral channels, nominal and measured frame rates, timestamps, exposure and gain controls, white-balance policy, image resolution, face/skin-region size, illumination, and encoding settings. These are proposed reporting requirements, not a universal hardware specification.

Temporal sampling requirements depend on the intended output. At 30 frames/s, successive frames are approximately 33.3 ms apart; at 60 frames/s, they are 16.7 ms apart. Estimating a dominant pulse frequency over several seconds is less demanding than locating individual pulse peaks for interval variability. Interpolation can refine an estimator but does not restore information absent from the acquisition. Actual timestamps and dropped frames should be retained. Mironenko et al. found that the frame rate irregularity tested in their experiments did not substantially distort pulse spectra, while rolling-shutter acquisition introduced spatial phase shifts of up to approximately 20 ms [20]. This distinction matters: an apparently accurate average HR estimate does not establish that inter-region phase differences represent physiological transit times. The window length must also accompany any accuracy claim.

Exposure, white balance, and illumination require explicit control or characterisation. Automatic gain or colour channel adjustments can change the measured temporal trace, but disabling all automatic control is not invariably optimal when lighting changes. Work on dynamic exposure control and suppression demonstrates that acquisition and processing can be designed jointly [21]. For reproducible experiments, a stable, nonsaturating illumination arrangement and documented camera settings are preferable; evaluation should then introduce realistic lighting and motion changes deliberately. Spatial resolution should likewise be reported together with the number of usable skin pixels, because image dimensions alone do not describe the physiological signal available after face cropping.

Compression is an experimentally demonstrated domain shift. Nowara et al. evaluated five compression levels and three encoding formats, using participant-independent five-fold validation on 25 participants [22]. In the stationary condition, increasing the constant rate factor from 12 to 30 raised the HR MAE of a model trained at CRF 12 from 1.7 to 9.5 bpm; training at the matching CRF reduced the latter error to 3.5 bpm. Even matching training did not remove severe degradation: MAE reached 8.0 bpm at CRF 36. These settings are experiment-specific, not universal bitrate thresholds. Telehealth evaluations should document the complete transmission/encoding path and test transmitted video rather than infer suitability from uncompressed laboratory recordings.

Alternative sensors change the available evidence. Near-infrared imaging can support pulse measurement beyond visible-light acquisition, but its spectral response requires adapted methods [23]. Thermal imaging measures emitted radiation and regional temperature dynamics rather than RGB colour pulsatility. In a pilot involving 15 men, Engert et al. found stress-sensitive facial temperature changes, especially around the nose, but thermal patterns did not reliably distinguish anticipation, stress, and recovery [24]. This is evidence for context-dependent autonomic information, not a disorder-specific thermal signature. Depth-based approaches have estimated HR and rhythm from three-dimensional motion [25]. Event-camera feasibility has also been demonstrated, including wrist recordings from 25 participants [26]. Neither result establishes facial psychiatric screening validity. Depth and event sensing should therefore be treated as additional research modalities whose incremental value, acquisition burden, and clinical validation remain to be established for the tasks reviewed here.

### 3.2. Face Localisation, Alignment, and Measurement Quality

Face detection locates a face, whereas landmark estimation and tracking assign facial coordinates across time. MTCNN combines detection and alignment through cascaded networks [27]; RetinaFace jointly predicts face boxes and facial geometry [28]. The commonly used dlib 68-point shape predictor implements an ensemble of regression trees [29]; it should not be confused with the detector that supplies its input face box. The original MediaPipe face-mesh model estimates 468 vertices [30]. Its iris-refined configuration adds ten iris landmarks, producing 478 points [31]; the model configuration should therefore accompany any stated landmark count. Monocular mesh depth is inferred geometry, not an independent calibrated depth measurement. Three-dimensional morphable models provide another route to fitting facial shape and appearance [32].

Preprocessing choices define what subsequent features can measure. Alignment can reduce camera-relative translation and rotation, but removing rigid motion also removes head movement that may itself be an analysis target. A suitable pipeline therefore preserves pose trajectories separately from aligned appearance. Detection confidence, visible-face duration, occlusion, and interpolation should be documented. Excluding low-quality frames changes the observations entering a participant’s prediction; if exclusion is associated with symptom severity or recording setting, this can introduce selection bias. Cropping and resampling also alter spatial detail, while colour processing may affect the physiological signal. These considerations connect the front end directly to the acquisition and confounding issues discussed below.

### 3.3. Behavioural Features and Micro-Expression Resources

Landmark trajectories, head pose, gaze estimates, and facial action units (AUs) provide different descriptions of behaviour. OpenFace 2.0 offers landmark tracking, head-pose estimation, AU recognition, and gaze estimation in a common toolkit [33]. Estimated AU occurrence or intensity can support interpretable summaries, such as movement frequency or duration, but these outputs remain algorithmic measurements with their own error. An AU is a description of facial movement, not a psychiatric category. Speech, interaction context, fatigue, and recording quality must be considered when interpreting apparent differences between groups; disorder-specific conclusions require evidence beyond a feature’s predictive association.

Handcrafted appearance descriptors remain useful reference representations. LBP-TOP encodes texture variation on three orthogonal planes, explicitly combining spatial and temporal information [34]. Its temporal neighbourhood, scale, and alignment must be reported: a descriptor calculated over a few frames is not equivalent to a summary of an entire interview. A comparison with a deep model is meaningful only when both receive comparable observations and use the same participant partition.

Micro-expression terminology requires particular care. CASME II contains 247 annotated clips from 26 participants recorded at 200 frames/s [35]; SAMM contains 159 micro-movement samples from 32 participants and also uses high-frame-rate acquisition [36]. CASME II is distinct from CAS(ME)^2^ and other versions. Duration criteria are operational conventions: CASME II selects movements with total duration below 500 ms or onset duration below 250 ms [35]. Neither resource supplies a psychiatric reference standard. They support facial-motion method development, while any contribution to mental health screening requires separate clinical validation.

### 3.4. Learned Spatial, Temporal, and Relational Representations

VGG-Face, ResNet, and EfficientNet provide representative spatial encoders [37,38,39], but their original recognition tasks do not establish clinical validity. A frame encoder can supply features to a temporal model; frame averaging alone discards ordering. Three-dimensional convolution learns local spatiotemporal patterns, illustrated by C3D and I3D [40,41]. Video transformers instead operate on sequences of spatial or spatiotemporal tokens, with factorised variants trading interaction range against computation [42]. Neither architecture guarantees that a model trained on short clips captures a complete interview.

Graph-based representations make relationships explicit. ME-GraphAU learns AU node features and sample-dependent edge features and evaluates AU recognition on BP4D and DISFA [43]. This supplies a concrete entry point for relational facial modelling, rather than evidence of improved depression screening. Similarly, VideoMAE demonstrates masked video pretraining [44], and MARLIN adapts reconstruction to facial regions and evaluates transfer across facial-analysis tasks [45]. Their results motivate investigating pretraining but do not establish mental health benefits. Participant-disjoint evaluation, documented pretraining exposure, and matched ablations are needed to separate useful behavioural transfer from identity or acquisition shortcuts. Table 2 compares these modelling assumptions and their application-specific limitations.

### 3.5. Remote Photoplethysmography: Algorithms, Evidence, and Interpretation

**Figure 3:**
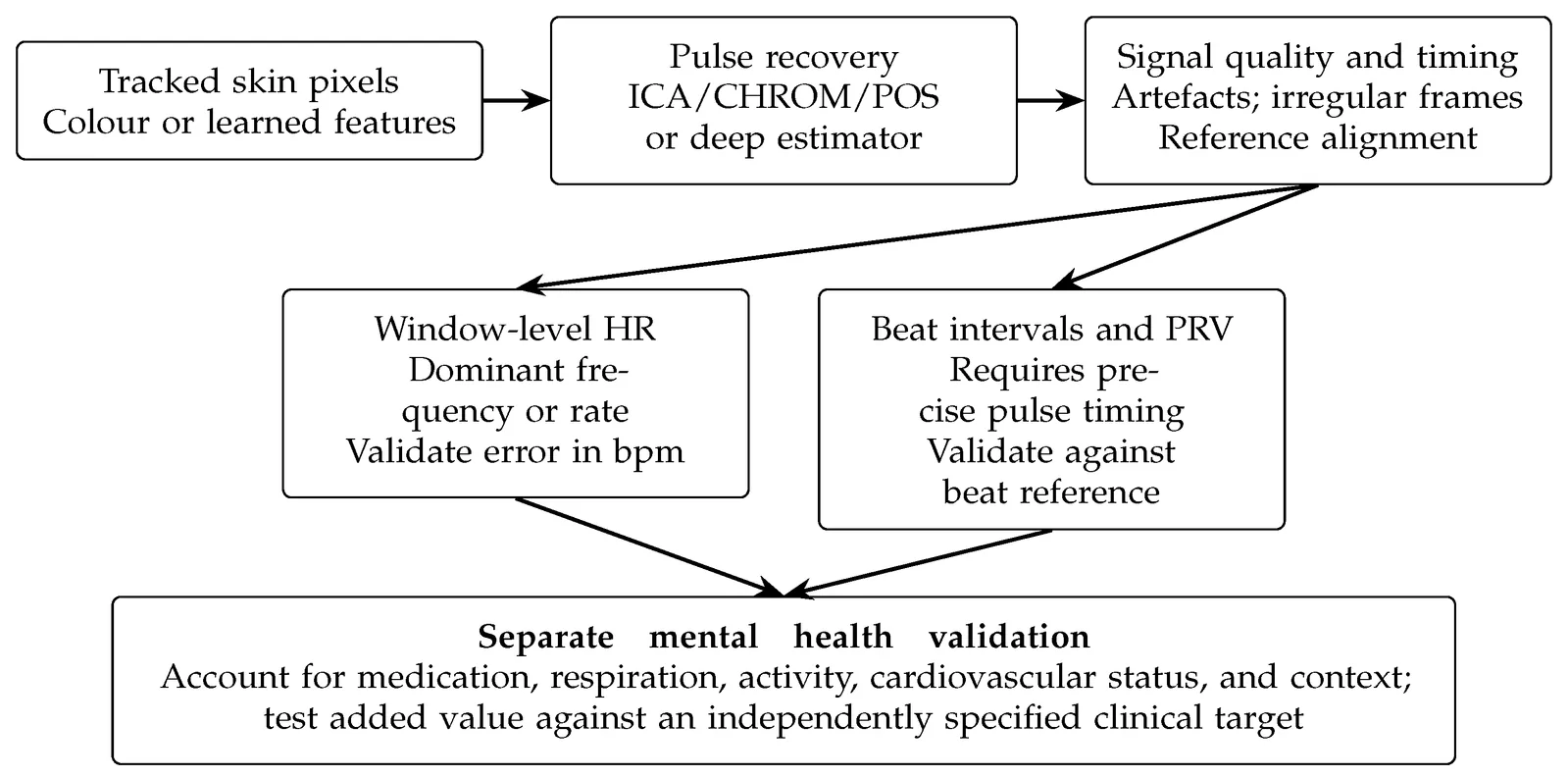
rPPG signal chain and its two validation boundaries. Low error in average HR does not imply accurate beat intervals, and accurate physiology does not imply disorder specificity. A single facial optical trace does not independently provide pulse transit time.

An rPPG pipeline tracks visible skin, extracts colour traces or learned video features, suppresses nuisance variation, and estimates a pulsatile waveform. Measurement quality must be checked before deriving physiological summaries or supplying them to a mental health model. Figure 3 separates these stages to emphasise that successful downstream prediction cannot, by itself, validate the intermediate physiological measurement.

**Classical and learned estimators.** Poh et al. combined face tracking with independent-component analysis of colour channels [47]. CHROM uses chrominance combinations to improve robustness to movement, while POS projects temporally normalised RGB signals onto a plane orthogonal to the skin-tone direction [19,48]. These methods provide inexpensive, inspectable baselines; their assumptions can fail when movement, illumination changes, or encoding artefacts dominate the weak pulse component.

DeepPhys learns physiological changes from normalised frame differences, using an appearance stream to guide spatial attention [49]. PhysNet uses spatio-temporal networks to reconstruct a pulse waveform and explicitly targets information beyond average HR [50]. RhythmNet encodes colour changes across multiple facial regions into spatio-temporal maps and combines convolutional features with recurrent temporal modelling [51]. PhysFormer uses temporal difference operations and attention to model local changes and longer-range periodic relationships [52]. These designs represent different allocations of prior knowledge and learned processing; deeper temporal modelling does not guarantee robustness to an unseen device or population. Table 3 summarises representative HR-estimation results under the reported benchmark protocol.

Self-supervision reduces reliance on synchronised contact labels but introduces its own assumptions. Contrast-Phys learns from spectral consistency within a video and differences between videos [53]. Such consistency is useful when multiple facial regions share a physiological source. However, periodic illumination or movement may also create coherent signals; this is a methodological failure mode to test, rather than a claim that an unsupervised objective necessarily identifies the cardiac source. Contact-reference evaluation remains necessary, including waveform quality and interval accuracy when those outputs are used.

Within this benchmark, PhysFormer reduced MAE by 0.33 bpm, approximately 6.2%, relative to RhythmNet, while the difference from earlier end-to-end models was larger. This comparison concerns physiological measurement under the stated protocol (Box 1). VIPL-HR acquisition variation and MMSE-HR are described in Section 5.1. Waveform/variability evaluation additionally requires appropriate synchronised references and recording durations.

**HR, pulse rate variability, and ECG-derived HRV.** HR summarises cardiac frequency; pulse rate variability (PRV) summarises intervals between peripheral pulse landmarks; ECG-derived HRV uses cardiac R–R intervals. These are related but nonidentical quantities. In an experimental cold-exposure study, Mejía-Mejía et al. showed that PRV and HRV differed according to condition and measurement site [54]. For facial video, sampling, pulse-peak localisation, vascular dynamics, and artefact handling add further uncertainty. Accordingly, camera-derived standard deviation of normal-to-normal intervals (SDNN) or root mean square of successive interval differences (RMSSD) should be identified as PRV-derived estimates unless interchangeability with ECG has been demonstrated for that setting. Low HR MAE alone does not validate these indices. Medication, activity, sleep, cardiovascular status, and situational arousal should be treated as potential confounders in the clinical study design, not assigned a psychiatric interpretation solely from a pulse trace.

**Direct symptom and stress evidence.** The depression-scale rPPG evidence extracted here comes from the following study; the stress studies discussed below address different endpoints. Álvarez Casado et al. studied BDI-II severity regression on AVEC2013/2014, rather than clinician-confirmed case–control diagnosis [55]. On AVEC2014, pooling Freeform and Northwind tasks, their rPPG-only random forest model achieved MAE/RMSE of 7.44/9.55 BDI-II points. Combining physiological, textural, and deep visual predictions yielded 6.57/8.49. However, the deep-only model achieved 6.83/9.06, and adding rPPG to that stream alone gave 6.90/8.88: benefit depended on the metric and fusion configuration. Physiological features therefore appear potentially complementary, rather than uniformly advantageous or diagnostic. The study also used six-second feature windows; physiological interpretation of long-period variability descriptors warrants particular caution at that duration.

Skin-tone robustness requires explicit subgroup testing. Nowara et al. reported that inverse-attention denoising reduced HR MAE in the MMSE-HR skin-type-VI subgroup from 8.77 to 1.57 bpm relative to their convolutional attention comparator [56]. This is a useful mitigation result within that experiment, not evidence of demographic parity or transfer to mental health cohorts. Reports should include subgroup sample sizes, uncertainty, signal-quality rejection rates, and the interaction of lighting with skin appearance. Otherwise, quality-based exclusion can hide unequal measurement failure.

Frame alignment has also been applied to webcam imaging photoplethysmography (iPPG) for experimentally induced stress recognition [57]. Section 5.2 records the controlled setting and unresolved participant grouping that limit interpretation of its high reported accuracy.

UBFC-Phys provides an additional bridge from physiological recovery to experimental stress recognition, with facial video, contact blood-volume pulse and electrodermal activity [58]. Xu et al.’s multi-task model uses an rPPG peak-attention mechanism and auxiliary heart rate learning on this dataset [59]. It reports 94.33% accuracy for stress state and 83.83% for stress level; Section 5.2 preserves the unresolved participant partition detail. These are elicited stress endpoints, not prospective anxiety disorder screening. Thus, the evidence reviewed here extends beyond a single rPPG study, while remaining limited for depression-specific inference and independent clinical deployment.

## 4. Learning Paradigms and Architecture Trade-Offs

### 4.1. Targets and Temporal Aggregation

The target should be specified before choosing an architecture. Binary prediction of a scale cut-off, regression of a symptom score, and recognition of a stress induction condition optimise different objectives. An AU or emotion prediction auxiliary task may regularise a representation under the target distinctions in Box 1. In a long interview, a participant-level score is often attached to many short clips. Those clips provide repeated observations of the same target; treating them as independent labelled people exaggerates the effective sample size.

Aggregation is part of the model. Mean pooling is invariant to observation order and assigns equal weight to the retained clips; attention pooling can emphasise a small number of segments but may also select identity, interviewer behaviour, or lighting artefacts. Recurrent or temporal–convolutional models represent ordering explicitly, whereas unordered frame averaging cannot identify whether a movement was sustained, repeated, or brief. Evaluation must therefore describe clip length, sampling, the handling of failed face tracks, and the rule that converts clips into one session or participant prediction.

### 4.2. What Architectural Differences Can Explain

Table 2 compares modelling assumptions, not a performance hierarchy. A pretrained 2D encoder with a modest temporal head can be a reasonable reference model when independent participants are scarce. A 3D convolution offers a local temporal prior, but its receptive field and temporal downsampling determine which movements survive. A transformer can connect distant tokens within its input, yet a model trained only on short crops does not automatically learn interview-scale context. Graph models restrict interactions through a chosen topology; this can reduce complexity while transmitting landmark errors through connected nodes. Interpretability is not guaranteed by attention weights or graph structure.

These distinctions motivate controlled ablations. To attribute a gain to temporal attention, studies should hold the participant split, backbone pretraining, input duration, augmentation, and optimisation budget fixed. To attribute a gain to facial physiology, the same visual model should be compared with and without that channel under matched acquisition and labels. A comparison in which architecture, modality, pretraining corpus, and split all change cannot identify which component caused the improvement. The matched examples in Section 5.2 are more informative than a chronological ranking assembled from unrelated scores.

Bargshady and Goecke investigate image and video vision transformers for depression severity estimation on AVEC 2013/2014 [60]. The study provides a facial video example of temporal attention, while a matched comparison still requires the same pretraining and evaluation split.

Recent hybrids illustrate these choices without establishing a universal winner. LMTformer combines multiscale temporal processing with a lightweight transformer, while LOGLformer combines local and global facial characteristics; the latter evaluates AVEC 2013/2014, not AVEC 2019 [61,62]. WavFace combines audio and facial features on DAIC-WOZ [63]. Available results and the limits of their interpretation are recorded explicitly in Section 5.2; none is assigned an assumed participant-independent protocol or used to construct a quantitative ranking. MMFformer reports ten-fold results on vlog datasets [64]; the available grouping and metric information does not establish comparability with the original fixed-split D-Vlog benchmark. A high published score is therefore insufficient evidence of a matched improvement.

### 4.3. Multimodal Fusion and Missing Information

**Figure 4:**
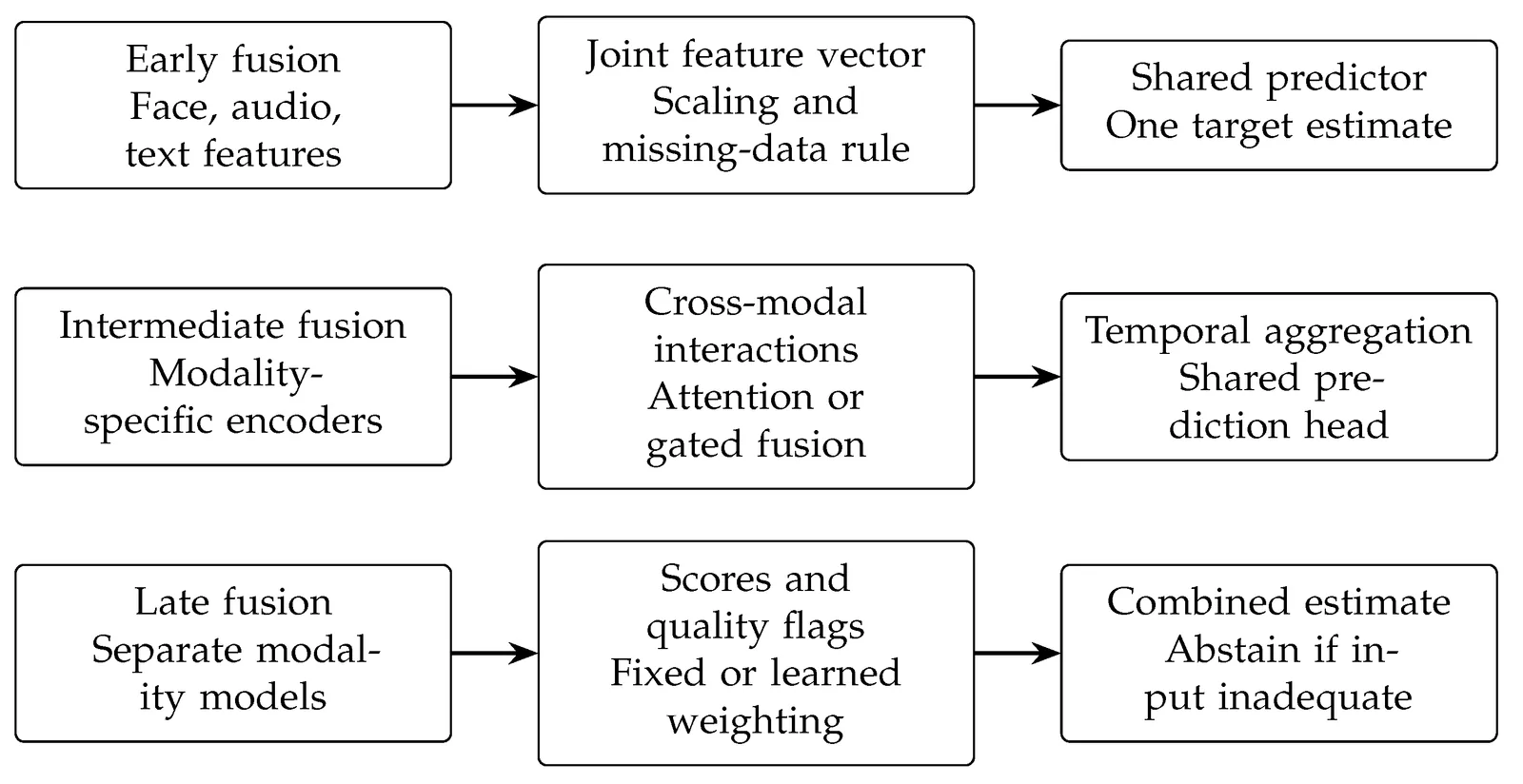
Three multimodal fusion designs. The comparison concerns where information is combined; it does not imply that one design is always more accurate. All designs need matched unimodal baselines and evaluation of missing or unreliable channels.

Figure 4 distinguishes the points at which modalities interact. Early fusion concatenates features and learns their joint representation; intermediate fusion permits interactions between modality-specific encoders; late fusion combines separately estimated scores. Early fusion is simple but sensitive to feature scaling and absent channels. Cross-attention can model alignment when the streams are synchronised, while introducing more parameters and opportunities to learn dataset-specific correlations. Late fusion can preserve separate quality estimates, but it may discard useful timing relationships.

In clinical interviews, language can contain direct symptom reports or question–answer structure. A high multimodal score therefore does not demonstrate that facial behaviour is a strong independent signal. Facial incremental value should be measured against audio-only, text-only, and audio-text baselines on the same people. Missing-channel evaluation should cover realistic failures—poor face visibility, corrupted audio, absent transcripts—instead of only random feature masking. Modality-specific quality indicators and an abstention mechanism are especially relevant when a fused model would otherwise make a confident prediction from unreliable input.

### 4.4. Pretraining, Data Scarcity, and Explanations

Transfer learning, masked video modelling, and affect-pretrained encoders can reduce the amount of task-specific supervision needed, but transfer is an empirical hypothesis. A representation that separates expression categories may retain identity, demographic, or acquisition information that is unhelpful for symptom estimation. Pretraining overlap with benchmark participants must be checked where possible. Augmentation should preserve the intended signal: colour perturbation can destroy rPPG, and temporal rearrangement can remove precisely the dynamics being studied.

Attribution should be tested as a measurement rather than accepted because an explanation appears plausible. Perturbation sensitivity, stability across seeds, and agreement with independently coded behaviours can provide checks. An AU attribution shows that the fitted model uses that estimated feature; it does not establish an AU as a disorder-specific or causal biomarker. A small model with transparent inputs and calibrated participant-level predictions may offer a stronger deployment baseline than a larger model with an unvalidated explanation.

Expresso-AI supplies a concrete example of attribution revealing a potential shortcut: its authors observed substantial relevance on headsets and microphones in AVEC 2014 videos [65]. Thus, an explanation can expose recording-associated cues rather than confirm a clinical mechanism. Its R(2+1)D result and training/development combination are reported in Section 5.2; the source is an AVEC 2014 study, not a DAIC-WOZ experiment.

## 5. Datasets and Evaluation

### 5.1. Datasets: Population, Labels, and Appropriate Use

Dataset size must be expressed in independent people, recording sessions, and derived frames or segments separately. Thousands of frames from one interview do not provide thousands of independent clinical observations. Table 4, Table 5 and Table 6 distinguish questionnaire-linked resources, social media proxy labels, and supporting affect/physiology benchmarks. Here, the term “mental health labels” describes the recorded reference variable; it does not imply clinician-confirmed psychiatric diagnosis.

These resources illustrate why a general claim that clinical datasets contain fewer than 300 participants is too broad. DAIC-WOZ, E-DAIC, and the AVEC 2014 participant pool are small, whereas the school-based FACES cohort is substantially larger but has a different population and target definition [16,67,68,69]. The relevant sample size for uncertainty is the number of independent held-out participants, not the number of video frames. FacePsy also illustrates a different deployment task: repeated smartphone observations predict questionnaire-defined periods with a study-specific threshold [70]. It should not be presented as a crowdsourced social media AU corpus.

Social media sampling offers more diverse recording environments but changes the inference problem. A model may learn presentation style, disclosure vocabulary, recording context, or platform-related cues associated with the annotation process. D-Vlog labels were assigned from vlog content rather than administering PHQ-9 [71]; therefore “PHQ-9 proxy” is an inaccurate dataset description. LMVD similarly requires its annotation procedure to be distinguished from prospective participant assessment [72]. For both resources, results should be qualified by label provenance, repeated identities, platform composition, and the intended population. Cross-dataset testing needs to consider label mismatch as well as visual domain shift.

Affect pretraining can improve feature initialisation without supplying evidence that a representation measures a psychiatric condition. Expression categories, AU occurrence, and valence/arousal are different targets from PHQ-8 or BDI-II scores [73,74,75,76]. PURE and UBFC-rPPG provide physiological references for the first validation layer in this review’s Box 1 [77,78]. MAHNOB-HCI includes affective elicitation and physiological recordings, so its role must be specified at the level of the experiment and retained participants [81]. PURE was introduced at RO-MAN 2014; using a consistent publication year convention avoids confusing dataset introduction, online publication, and journal issue dates.

Finally, “public dataset” should not imply unrestricted biometric access. DAIC resources use an application process; some social media resources release derived features; FACES directs users to its project for accessibility updates [16,67,71,72]. Replication reports should state the release, access agreement, available input representation, exclusions, identity grouping, and final train/development/test counts. These details determine what architecture can actually be reproduced and what population its reported performance describes.

### 5.2. Quantitative Evidence and Its Comparability

Table 7 uses local Gabor binary patterns from three orthogonal planes (LGBP-TOP), Moments in Time (MiT) pretraining, the concordance correlation coefficient (CCC), and unweighted average recall (UAR). These names identify features, pretraining, or metrics rather than additional clinical targets.

What the quantitative evidence supports.

Box 1 provides the interpretation rules for Table 7. The official AVEC 2014 protocol guarantees separation of sessions, but the challenge description does not establish participant separation across repeated sessions [69]. A stronger participant-independent claim requires identity-level verification.

Within-study ablations offer more defensible explanations of improvement. In the global/local C3D study, the AVEC 2014 global-face baseline achieved MAE 7.23 and RMSE 8.99; combining full-face and eye-region branches with global-average pooling yielded 6.59 and 8.31, respectively [83]. The corresponding reductions are 0.64 MAE points and 0.68 RMSE points (8.9% and 7.6%). This supports complementary regional information and a compact pooling operation under that experiment. It does not isolate every implementation change or establish that an eye-region representation is a disorder-specific biomarker.

Temporal aggregation also changes the result independently of the backbone. ExpADA’s within-paper Freeform ablation reports MAE/RMSE of 7.40/9.26 for base regression, 6.51/8.10 with ordinal supervision, and 5.83/7.64 with relevance-based feature selection [85]. The complete change reduces MAE by 1.57 and RMSE by 1.62 points. Its overall AVEC 2014 result of 5.11/6.89 additionally fuses predictions across Freeform and Northwind. Reporting only that final pair would conceal an acquisition-task and aggregation difference. The authors also explicitly distinguish their highlighted segments from expert-validated clinical markers.

Different metrics can favor different systems even within one benchmark. On the same E-DAIC test partition, the AVEC 2019 audiovisual fusion baseline reduced RMSE from the visual ResNet baseline’s 8.01 to 6.37, a 1.64-point reduction (20.5%), while CCC declined from 0.120 to 0.111 [68]. Lower average scale error therefore did not imply improved concordance. Reporting both measures is more informative than selecting a favorable metric, particularly when prediction shrinkage toward the mean can reduce error while missing between-person variation.

For vlog classification, the original D-Vlog paper reports weighted F1 of 59.70% for its BLSTM comparator and 63.50% for its cross-attention model, a 3.80-percentage-point difference under the same channel-disjoint partitions [71]. Its cross-corpus experiment obtained weighted F1 of 57.73% when training on D-Vlog and evaluating on DAIC-WOZ. However, that experiment used a custom 112/37/38-sample DAIC-WOZ partition, so it cannot be compared directly with official challenge results. DepMSTAT’s reported F1 of 73.51% is retained with an explicit averaging caveat, rather than converted into an unconditional improvement over the earlier weighted-F1 result [86].

Uncertainty and validation coverage remain uneven. DNet reports intervals across repeated random seeds, but its repeated-run means differ from its headline results [84]. Such intervals describe training variability under a fixed dataset; they are not participant-level confidence intervals for clinical generalization. Evaluation on another dataset after separate training is also different from applying a frozen model to an external population. These distinctions constrain claims about progress: controlled architectural gains are useful, but prospective performance, calibration, demographic robustness, and clinical utility remain separate questions.

FACES supplies a quantitative anchor for adolescent anxiety and stress [16]. The unfiltered and filtered rows reproduce standard binary classification from Table V of the author version (arXiv:2405.20072v2); they do not use the separate “refined” task that also removes mild cases. Filtering changes the population, so the increased scores cannot be attributed to a better architecture. The extracted reporting leaves the F1 averaging and nesting of data-selection decisions unresolved. These features limit deployment extrapolation despite the comparatively large cohort.

Table 8 records additional methods whose reporting limits a matched quantitative comparison.

The high reported stress classification accuracy was obtained under controlled webcam acquisition and an arithmetic stressor, not a clinical anxiety reference. Randomised folds without an explicit participant grouping cannot establish generalisation to new users [57]. Likewise, architecture names and headline F1 values cannot resolve differences in label provenance, grouping or metric averaging. Recording these limitations is preferable to silently filling missing protocol fields.

### 5.3. Participant-Level Validation and Uncertainty

All frames, overlapping windows, augmented clips, and repeated sessions from one participant should remain on the same side of a participant-independent split. For social media corpora, uploader or channel identity is a useful grouping variable, but it is not proof that every person appears in only one channel. Official challenge partitions must be described as published: session-disjointness should not be silently relabelled participant-disjointness. The number of independent people and the number of positive cases determine uncertainty more directly than the number of extracted frames.

**Figure 5:**
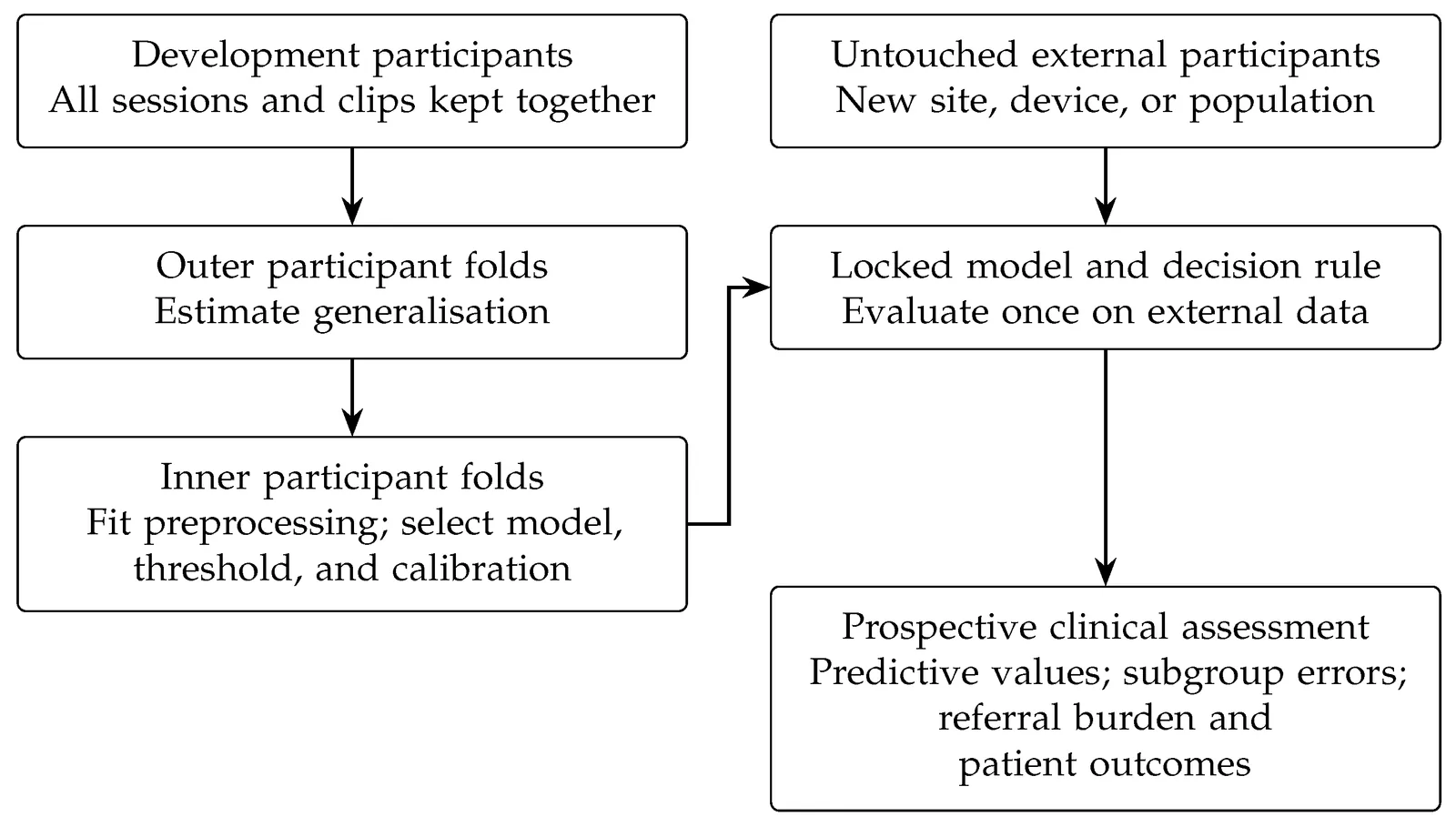
Participant-level development and translation. Inner validation is contained within each outer training partition. The schematic separates internal model selection, external testing, and prospective assessment; success at an earlier stage is not a substitute for a later one.

Where model and threshold selection require cross-validation, an outer participant split estimates generalisation and inner participant splits select preprocessing, features, hyperparameters, calibration, and the operating threshold [87]. Standardisation, feature selection, oversampling, and data-driven dimensionality reduction must be fitted within the relevant training partition. A locked external test set should remain untouched during these choices. Figure 5 distinguishes these stages from prospective evaluation. The final fitted model, test–time aggregation, failed-acquisition exclusions, and missing-data rules need to be stated together [88].

Classification reports should identify the positive class and distinguish positive-class, macro, micro, and support-weighted F1. Accuracy is insufficient under imbalance; sensitivity, specificity, confusion matrices, and precision–recall performance help to describe the error trade-off. ROC AUC measures ranking over thresholds, not the predictive value of one deployment rule. Regression should report the scale and its range, MAE and/or RMSE, and where useful calibration of the predicted severity. Correlation alone can remain high despite systematic score offsets; concordance coefficients address a different property and are not interchangeable with Pearson correlation.

Uncertainty should match the unit of inference. Participant-level bootstrap intervals can describe test population uncertainty, whereas repeated random-seed summaries describe training variability conditional on the data. They are not substitutes for one another. Frame-wise resampling is inappropriate when frames are clustered within participants. Paired resampling of participant predictions is preferable for a within-test set model comparison; a small numerical difference should not be called significant without a suitable paired analysis. Most extracted benchmark results do not provide a verified participant-level interval, and this is a limitation of the evidence available for synthesis.

Cross-dataset testing introduces simultaneous changes in language, population, recording, target distribution, and label definition. Performance loss should be analysed along those axes instead of as a single generic “domain gap”. External evaluation should preserve the intended target, or explicitly describe any label mapping. Site, device, demographic group, and relevant acquisition quality strata should be reported with denominators and uncertainty. A subgroup average without enough positive cases cannot establish equivalent safety.

### 5.4. Prevalence, Thresholds, and Clinical Interpretation

A screening instrument operates at a particular threshold in a particular population. For sensitivity Se, specificity Sp, and target prevalence π, the positive and negative predictive values are

(1) $$\mathrm{PPV}=\frac{\mathrm{Se}\pi }{\mathrm{Se}\pi +(1-\mathrm{Sp})(1-\pi )},\;\mathrm{NPV}=\frac{\mathrm{Sp}(1-\pi )}{\left(1-\mathrm{Se})\pi +\mathrm{Sp}(1-\pi \right)}.$$

As an illustrative calculation, not an empirical result, suppose sensitivity and specificity both equal 0.80. At a prevalence of 5%, PPV is 17.4% and NPV is 98.7%; at 20%, PPV is 50.0% and NPV is 94.1%. The calculation assumes those sensitivity and specificity values transfer unchanged. In practice, case mix and acquisition conditions may change them. It shows why an apparently useful balanced-corpus result can still generate a large referral burden in a low-prevalence setting. If the target is questionnaire positivity, the prevalence in this equation is the prevalence of that label, not automatically the prevalence of a diagnosed disorder.

Thresholds should reflect the intended follow-up pathway, the relative consequences of missed cases and false alarms, and available assessment capacity. They should be selected without using test outcomes. Calibration assesses whether predicted probabilities correspond to observed outcome frequencies in the target population; reliability plots, calibration intercept/slope, and proper scoring rules can complement discrimination metrics. Any local recalibration requires a separate evaluation after fitting. A model may also require a quality-based reject option when the face track or physiological signal is inadequate.

A questionnaire cut-off is an imperfect reference for a clinical disorder. Agreement with it measures reproduction of its operational definition. Its sensitivity and specificity against diagnostic interviews do not impose a universal mathematical upper bound on a model’s agreement with the questionnaire labels themselves: a model could reproduce those labels while sharing their systematic errors. Clinical validity instead requires comparison with an independently established reference, clear timing between measurements, and evaluation of downstream consequences. The PHQ-9 diagnostic-accuracy evidence should therefore guide interpretation, rather than be used to relabel scale prediction as diagnosis [10].

## 6. Application Scenarios and Evidence Requirements

### 6.1. Teleconsultation and Supported Screening

A useful distinction is between collecting video and analysing the face. Grimm et al.’s single-question assessment uses text and voice models to predict PHQ-9, GAD-7 and PCL-5 scores [89]. It informs remote-assessment workflow; facial incremental value needs a separate comparison. A plausible use of facial analysis is to support an already-consented clinical interaction, with the model output reviewed alongside the interview and questionnaire. The system would need to explain what target it estimates, whether input quality was adequate, and what follow-up action is appropriate. Current benchmark evidence does not establish improved patient outcomes or reduced clinician workload. These must be measured prospectively, including time spent resolving false alerts and whether a score changes clinical decisions appropriately.

Di Lernia et al. tested browser-based webcam rPPG in an online study with 18 participants, using a finger-camera smartphone measurement as the remote heart rate reference [90]. Their acquisition and quality filtering support online measurement feasibility under the tested conditions; the endpoint was heart rate. Teleconsultation also couples two sensing problems. A compressed call may preserve recognisable facial expressions while attenuating small pulse-related colour variations. The viability of behavioural analysis and rPPG should therefore be evaluated separately on the actual end-to-end call path, including camera settings, codec, network adaptation, and dropped frames [22]. A valid result from a locally recorded video is not sufficient validation of a conferencing implementation.

### 6.2. Personal and Longitudinal Monitoring

Naturalistic smartphone collection differs from a fixed-camera interview: capture opportunities depend on phone use, pose, ambient light, and participant willingness. Missingness can be related to symptoms or daily routines. A model may consequently learn engagement with the device rather than symptom change. Analyses should report usable recordings and missing days per person and evaluate whether the relationship persists after accounting for these patterns.

Plevin et al. synthesised 12 studies of facial expression and response to depression treatment [8]. Improvement was generally associated with greater facial activity, but methods differed and small samples, limited blinding, and sparse repeated measurements constrained interpretation. This supports further longitudinal investigation while keeping pretreatment response prediction distinct from monitoring change during treatment.

Personal baselines can help to distinguish within-person change from stable between-person differences but require enough observations to estimate ordinary variability. A monitoring model should be evaluated on future sessions and, where the intended use includes new users, on held-out people. Treatment response prediction, concurrent symptom monitoring, and forecasting future deterioration are separate endpoints. Prospective evaluation must avoid deriving a feature from observations collected after the outcome it is intended to predict.

### 6.3. Social Media and In-the-Wild Research

BHDD models low-level facial behaviour and hierarchical multimodal fusion on vlog data [91]. Its author implementation provides pipelines for LMVD and D-Vlog, illustrating how landmarks, action units, pose, and gaze can complement audio.

Vlog datasets broaden recording conditions but often use self-disclosure or other proxy labels rather than clinical assessment. Editing, topic, channel identity, and presentation style may explain part of a classifier’s performance. Public availability does not establish consent to psychiatric inference. These resources can support method research subject to their access and ethics conditions, but they do not justify individual risk assignment, unsolicited outreach, or workplace and insurance screening. Transfer to a clinical service requires a new consent process and independent validation against the intended reference standard.

### 6.4. Population-Specific Interpretation

School screening, inpatient assessment and community monitoring involve different populations and consequences. The FACES resource provides adolescent questionnaire targets, whereas a bipolar-state model addresses a condition-specific assessment [16,18]. An apparent gain in a narrowly recruited sample cannot establish performance in older adults, people with neurological comorbidity, or an unselected school population. These settings need appropriate consent, subgroup denominators, and a follow-up pathway. The number of recorded frames does not compensate for a lack of independent participants from the intended population.

## 7. Challenges in Generalisation, Ethics, and Deployment

### 7.1. Confounding and Transportability

The principal interpretive challenge is separating clinically relevant variation from participant identity, interview behaviour, treatment, and acquisition conditions. A model may exploit demographic or recording differences associated with the study labels, even when its predictions are accurate within the original cohort. Behavioural changes reported within a treatment study, for example, do not by themselves distinguish symptom effects from all effects of treatment or interview context [14]. Similarly, an rPPG estimator can measure physiology accurately without establishing that the measured variation is specific to a psychiatric condition.

Studies should report available medication, physical-activity, sleep and cardiovascular covariates, together with age, sex/gender, relevant population characteristics, and recording conditions. The purpose is to make alternative explanations testable. Depending on sample size and study design, useful checks include covariate-adjusted analyses, stratification, negative controls, and comparison with demographic-only or acquisition-only baselines. These analyses cannot resolve confounding from unmeasured variables, and small subgroup estimates require uncertainty intervals. Missing video or low signal quality may itself vary across participants; exclusion counts and reasons should accompany any analysis restricted to usable recordings.

Subject-independent evaluation must extend to preprocessing, feature selection, hyperparameter tuning, and calibration. Repeated sessions and overlapping clips from one participant should remain together when the target is performance on unseen participants. Nested validation separates parameter selection from performance estimation [87]; participant grouping adapts this principle to the dependence structure of facial video. External evaluation should additionally distinguish changes in clinical population, language, demographics, device, illumination and compression. A model adapted using target-domain data should be reported separately from an unchanged model tested on that domain. Reporting independent participant counts, outcome counts, performance uncertainty and data-handling procedures makes these claims assessable [88].

### 7.2. Consent, Secondary Use, and Potential Harms

Face recordings can reveal identity as well as health-related information. Consent should distinguish recording from mental health inference, model development, sharing, and future reuse. Data minimisation, access control and retention limits should cover derived embeddings and inferred labels alongside raw video. Removing names or replacing video with facial features should not be assumed to eliminate identification or inference risks. These concerns align with WHO guidance on protecting autonomy, privacy and accountability in health-related AI [92].

Public accessibility of a social media video does not by itself establish consent to psychiatric inference. Researchers should document the basis for secondary use, consider the context in which material was shared, and address withdrawal where feasible, and consider the risk of reidentification and the incidental capture of information about bystanders. The attraction of larger internet datasets must therefore be assessed against provenance, label quality, and participant expectations, not size alone. Collection from clinical care similarly requires a clear separation between receiving care and agreeing to optional research reuse.

Deployment evaluation should include false-positive distress and stigma, false reassurance after negative results, differential error across demographic groups, and inappropriate use by employers or insurers. A screening score should lead to an understandable, proportionate review or referral process, with a way to question the result. Human involvement must be meaningful: users need the intended scope, uncertainty, and known failure conditions, rather than an unexplained score. These systems are considered potential screening and decision support tools here, not autonomous diagnostic systems; benchmark performance does not establish deployment readiness.

### 7.3. Regulatory Scope Depends on Intended Use

Article 5(1)(f) of the EU AI Act prohibits AI emotion inference in workplaces and education institutions, with an exception for medical or safety purposes. Recital 18 distinguishes emotion inference from physical states such as pain or fatigue and from detecting expressions without inferring emotions [93]. The actual inference and intended use therefore matter: the provision should not be described as a blanket prohibition on all physiological monitoring or all mental health research. Calling an output “stress” is likewise insufficient to determine its legal classification without examining what the system infers and how it is used.

For medical-purpose software in the EU, qualification and risk classification follow the relevant Medical Device Regulation framework. MDCG 2019-11 rev.1 explains intended-purpose assessment and Rule 11, including software informing diagnostic or therapeutic decisions and monitoring physiological processes [94]. The resulting class depends on the use and consequences of error; this survey does not assign a single class to all reviewed systems. In the United States, FDA’s January 2026 clinical decision support guidance describes the criteria for the statutory non-device exclusion, including restrictions concerning medical image or acquired-signal analysis [95]. Describing software as “decision support” does not itself establish exemption, clearance or authorisation. A proposed product requires assessment of its specific functions, claims, and deployment setting.

## 8. Future Research Priorities

### 8.1. Foundation Models and Language-Assisted Systems

Large pretrained models may reduce annotation requirements or combine asynchronous signals, but their incremental value for facial mental health assessment remains an empirical question. An informative comparison includes a conventional model using the same modalities, a transcript-only baseline, and a small face video model under identical participant splits. Computation, calibration, robustness to missing channels, and the marginal contribution of video should accompany predictive performance. Benchmark overlap in pretraining and prompt tuning against test outcomes are additional leakage risks.

If a language model produces a narrative explanation, that explanation should be constrained to traceable observations and checked for unsupported clinical statements. Fluent text does not validate the underlying score or show that the model has recognised a psychiatric condition. Evidence of limitations in broader clinical decision tasks motivates these checks, while not directly measuring performance on facial screening [92,96]. Evaluation should include hallucinated symptoms, sensitivity to prompting, uncertainty communication, and whether readers place excessive trust in a verbal output. Retrieval or constrained output formats can limit some errors but cannot replace independent calibration and prospective evaluation.

SCD-MLLM provides a concrete attempt to combine heterogeneous inputs and handle missing modalities using input adapters and adaptive fusion [97]. Its accessible author manuscript describes joint training across five datasets. Evaluation of datasets represented during joint training must be distinguished from transfer to an entirely unseen clinical site or dataset. Such work motivates tests of missing-channel robustness, but comparison with selected commercial models does not alone establish calibrated probabilities, reliable explanations, or prospective clinical utility. This discussion uses the author manuscript identified in the reference for its training design, without extracting a numerical performance claim from that version.

### 8.2. Signal-Preserving Adaptation and Privacy

Domain adaptation should preserve the quantity being measured. An image transformation that improves apparent face quality can alter pulse chrominance or facial motion, so improvement in one downstream task cannot be assumed to transfer to another. Paired acquisition studies can separate hardware-induced errors from population effects; externally held-out sites and devices are needed to evaluate any adaptation procedure. Physiological recovery, behavioural measurement, and clinical prediction should each have their own endpoint.

On-device processing can reduce raw video transfer, while federated learning can limit central collection. Neither alone proves privacy: representations, updates, access logs, and stored outputs can still expose sensitive information. The research question is which data must be retained for a specified purpose, how access is controlled, and whether privacy protection preserves clinically relevant signal. Utility should be evaluated alongside the actual threat model rather than inferred from the name of the training method [92].

### 8.3. Prospective Evaluation of Incremental Benefit

The strongest next step is a preregistered, clinically specified evaluation, not merely a larger model. The population, comparator, reference standard, acquisition requirements, threshold, reject rule, and referral pathway should be set before outcomes are examined. Reporting should follow an explicit prediction-model framework [88]. A study should test whether facial sensing adds value beyond a questionnaire and routine information already available to the clinician. Appropriate endpoints include calibration, missed cases, unnecessary referrals, subgroup performance, workflow burden, and patient acceptability. A longitudinal early-warning claim additionally requires a prediction horizon and temporal separation between inputs and future outcomes.

## 9. Conclusions

Facial video supports several distinct measurements relevant to mental health research, including behavioural dynamics and camera-derived pulse signals. The results show technical promise within particular datasets and protocols. Box 1 summarises the interpretation principles that connect these findings to their intended use. Progress depends on transparent data provenance, participant-level evaluation, matched comparisons, acquisition-aware validation, calibration, and consent. Facial analysis should be developed as a possible complementary screening or decision support measurement whose added value and limitations are tested in the intended setting.

## Figures and Tables

**Table 1:** Positioning relative to selected prior reviews. Scope describes the principal emphasis; publication years do not identify search cut-off dates.

Review	Conditions/Modalities	Principal Evaluation Focus
Pampouchidou et al. (2019) [1]	Depression; visual behaviour	Visual cues and automatic depression assessment.
He et al. (2022) [2]	Depression; audio and video	Deep learning, objective cues and multimodal fusion.
Han et al. (2024) [3]	Depression; audio and video	Features, recognition methods and clinical application.
Tayarani-N. and Shahid (2025) [5]	Anxiety; multiple signal types	Machine-learning approaches to anxiety assessment.
Kyrou et al. (2025; online 2024) [6]	Stress; physiological and behavioural signals	Deep-learning stress detection across sensing modalities.
Khoo et al. (2024) [7]	Mental health; multimodal passive sensing	Sensing modalities, feature extraction and fusion approaches.
Plevin et al. (2026) [8]	Depression treatment; facial expression	Prediction and monitoring of treatment response.
Sakib et al. (2025) [4]	Contactless physiology; camera signals	rPPG methods and health-parameter measurement.
Present review	Depression, anxiety/stress and selected related tasks; facial behaviour and physiology	Acquisition constraints, reference labels, evaluation protocols and clinical interpretation, with separate rPPG measurement and mental health endpoints.

**Table 2:** Structural comparison of major model families. Benefits are modelling affordances, not a ranking of clinical performance. Generic vision or AU benchmarks do not establish mental health screening validity. All families require participant-level aggregation and evaluation.

Family	Structure and Potential Benefit	Limitation and Necessary Comparison
2D CNN [38,39]	Spatial filters encode each frame; established pretraining permits frozen or lightly adapted encoders.	No temporal ordering without an added component; compare aggregation rules and audit identity shortcuts.
CNN–LSTM	A recurrent LSTM state [46] integrates ordered frame features and accommodates variable sequence lengths.	Sequential processing and state compression; disclose truncation and compare identical input durations.
3D CNN [40,41]	Spatiotemporal kernels impose a local motion prior; inflated filters can reuse image pretraining.	Receptive field and temporal stride constrain visible dynamics; compare sampling, pretraining, and resource budgets.
Video transformer [42]	Attention connects tokens within the supplied temporal context; factorisation can reduce cost.	Dense attention cost grows quadratically with token count; subsampling may remove brief movements. Longer clips need separate validation.
GNN/AU graph [43]	Nodes and edges encode structural or learned relations; sample-dependent edges represent AU interactions.	Measurement errors propagate through relations; graph topology and AU accuracy require ablation. AU recognition is not clinical validation.
Multimodal fusion	Combines modality features, intermediate interactions, or predictions to use complementary observations.	Synchronisation and missing channels matter; compare audio-, text-, and vision-only baselines to establish facial incremental value.

**Table 3:** Representative HR-estimation results reported in Table 1 of the PhysFormer paper [52], for the VIPL-HR subject-exclusive five-fold protocol. Values are transcribed from that paper’s comparative table, not independently rerun. Differences in preprocessing, training, and implementation remain; this is not an architecture-only controlled experiment. These metrics assess physiological measurement and do not measure mental health screening accuracy.

Method	Representation	MAE (bpm)	RMSE (bpm)	Pearson *r*
CHROM [48]	Chrominance combinations	11.4	16.9	0.28
POS [19]	Skin-tone-orthogonal projection	11.5	17.2	0.30
DeepPhys [49]	Convolutional attention	11.0	13.8	0.11
PhysNet [50]	Spatio-temporal waveform reconstruction	10.8	14.8	0.20
RhythmNet [51]	Regional maps and recurrent modelling	5.30	8.14	0.76
PhysFormer [52]	Temporal-difference transformer	4.97	7.79	0.78

**Table 4:** Questionnaire-linked resources. Counts refer to the specified release or study. Interview acquisition does not convert a self-report score into a diagnostic reference standard. Access conditions and available modalities must be checked for each release.

Resource/Publication	People and Recordings	Target, Setting, and Qualification
DAIC-WOZ (2014) [66,67]	189 interview sessions in the distributed WOZ subset	PHQ-8 symptom scores (0–24); virtual interviewer controlled by a human. The provider distributes audio, transcripts and facial features; raw face video is not implied by feature access.
E-DAIC/AVEC 2019 DDS [68]	275 participants; train/development/test = 163/56/56	PHQ-8 severity; training/development mix human-controlled and autonomous interviews; test uses autonomous interviews. This is distinct from AVEC 2019’s state-of-mind task.
AVEC 2014 [69]	84 people; 150 sessions; 300 task clips	BDI-II (0–63); 150 Freeform and 150 Northwind clips. Sessions recur for some people. Official partitioning excludes session overlap; participant independence requires separate verification.
FacePsy (2024) [70]	25 completers; 544 days of facial data; 44 labelled two-week periods	Naturalistic smartphone facial sensing; repeated PHQ-9 (0–27). Binary periods require scores ≥5 at both endpoints, rather than a universal diagnostic threshold.
FACES (2026) [16]	11,427 recruited pupils; 8281 retained videos after quality screening	DASS-21 depression, anxiety and stress; ages 10–18; standardised reading videos. Questionnaire-defined symptom groups, not a diagnosed patient cohort; consult the authors for data-access status.

**Table 5:** Social media resources with content-derived proxy labels. These labels support research on observable/disclosed symptoms; they do not establish PHQ scores or verified psychiatric diagnoses.

Resource	Scale	Label Provenance and Access
D-Vlog (2022) [71]	961 vlogs; 816 speakers; approximately 160 h	Annotators interpreted disclosed current depressive symptoms in English YouTube videos; 555 positive and 406 negative vlogs. Released audiovisual features are de-identified. The original split separates YouTube channels.
LMVD (2026; online 2025) [72]	1823 vlogs; 1475 people; approximately 214 h	Binary content-derived depression labels across four social platforms; 908 positive and 915 negative samples in the author manuscript. The public resource provides features; raw facial video availability must not be assumed.

**Table 6:** Supporting affect/physiology datasets and stress-oriented resources. Stress-condition and self-rating labels are identified separately from physiological references; none is a psychiatric diagnostic reference standard. Years identify the cited publication; original release dates may be earlier.

Resource/Version	Scale	Supervision and Legitimate Role
*Affective representation learning*		
AffectNet [73]	>1 million retrieved images; approximately 450,000 manually annotated	Expression categories and valence/arousal; affect pretraining. Journal publication 2019; introduced in 2017.
RAF-DB (2017) [74]	29,672 images overall	Basic/compound expression labels; the actual subset is reported when comparing recognition results.
FERPlus (2016) [75]	35,887 source FER2013 entries reannotated	Label distributions from 10 raters per image; exclusions and label-processing rules change usable counts.
Aff-Wild2, BMVC 2019 version [76]	558 videos; 458 people; 2,786,201 frames	Valence/arousal, expressions and AU annotations, with task-specific subsets. Later ABAW releases have different sizes.
*Physiological measurement/affective elicitation*		
PURE (2014) [77]	10 people; 60 one-minute sequences	RGB video at 30 Hz and contact pulse reference; controlled motion protocols.
UBFC-rPPG, Dataset 2 [78]	42 released videos (of 46 recorded)	30 fps RGB and contact pulse-oximeter reference; mathematical task to elevate heart rate. Distinct from UBFC-Phys.
VIPL-HR [79]	107 people; 2378 RGB and 752 near-infrared videos	Heart rate references; variation in motion, illumination and camera devices. Physiological benchmarking.
MMSE-HR [80]	40 people; 102 videos	Facial video with physiological heart rate reference during spontaneous expressions; physiological robustness evaluation.
MAHNOB-HCI (2012) [81]	27 participants in the reported resource	Synchronized audiovisual, gaze and physiological recordings with elicited affect/self-ratings; subset exclusions must be reported.
*Stress-oriented facial and multimodal resources*		
UBFC-Phys [58]	56 people; 168 videos	Rest, speech and arithmetic tasks; two difficulty levels; facial video, wrist BVP/EDA and self-reported state anxiety. Distinct from UBFC-rPPG.
SWELL-KW [82]	25 people; about 3 h per person	Knowledge work under neutral, interruption and time-pressure conditions; facial expression features, posture, cardiac/skin-conductance signals and subjective ratings. Check access to raw video separately.
Giannakakis et al. study [15]	58 people; four phases and 11 tasks	Facial AUs under experimental stress/non-stress conditions; study cohort, with public raw video availability not established here.

**Table 7:** Representative primary-source results grouped by target and benchmark. Results are author-reported, not independently reproduced. BDI-II and PHQ-8 errors are in different questionnaire-point units; vlog classification uses a different reference standard. Official, session-disjoint, and channel-disjoint partitions are not interchangeable with verified participant independence. Numerical ranking across groups or different protocols is not justified.

Method	Dataset and Target	Input	Evaluation Protocol	Reported Performance
*Questionnaire symptom severity regression: AVEC 2014*				
LGBP-TOP + SVR (2014) [69]	AVEC 2014; BDI-II	Facial texture features	Official train/development/test; 50 session pairs per partition; session-disjoint	Test MAE 8.857; RMSE 10.859
Global/local C3D + 3D-GAP (2019) [83]	AVEC 2014; BDI-II	Full-face and eye-region video	Official test; video-level aggregation; participant disjointness not independently verified	Test MAE 6.59; RMSE 8.31
Expresso-AI (2023) [65]	AVEC 2014; BDI-II	R(2+1)D facial video	Training and development combined; official test; mean clip-score aggregation	MAE 6.626; RMSE 8.543; 50-layer, Kinetics-700 + MiT pretraining
DNet (2025) [84]	AVEC 2014; BDI-II	Global/local facial representations	Official train/development/test; participant disjointness not independently verified	Headline test MAE 6.09; RMSE 7.85 ^a^
ExpADA (2026) [85]	AVEC 2014; BDI-II	Facial video	Official test; decision fusion of Freeform and Northwind; participant disjointness not independently verified	Test MAE 5.11; RMSE 6.89 ^b^
*Questionnaire symptom severity regression: E-DAIC*				
AVEC 2019 visual baseline [68]	E-DAIC; PHQ-8	ResNet visual features	Official 163/56/56-subject train/development/test; autonomous-interviewer test recordings	Test RMSE 8.01; CCC 0.120
AVEC 2019 fusion baseline [68]	E-DAIC; PHQ-8	Audio and video	Same official partition as visual baseline	Test RMSE 6.37; CCC 0.111
*Adolescent questionnaire-defined anxiety and stress: FACES*				
Random forest, unfiltered [16]	FACES; DASS-21 anxiety/stress binary groups	Facial behaviour	Author version v2, Table V; five-fold CV; normal versus mild-or-higher labels; F1 averaging unspecified	Anxiety: accuracy 0.568, F1 0.546; stress: accuracy 0.570, F1 0.550
Random forest, 10% exclusion [16]	Same targets; filtered population	Facial behaviour	Same table; excludes 10% using authors’ inconsistency filter; changed sample composition, not matched-population gain	Anxiety: accuracy 0.626, F1 0.612; stress: accuracy 0.639, F1 0.620
*Classification of depression-related vlog labels*				
D-Vlog Depression Detector (2022) [71]	D-Vlog; binary content-assigned labels	Acoustic and facial landmark features	647/102/212-video train/validation/test; no shared YouTube channel	Weighted F1 63.50%; precision 65.40%; recall 65.57%
DepMSTAT (2024) [86]	D-Vlog; binary content-assigned labels	Acoustic and facial landmark features	Uses dataset train/validation/test partitions; exact channel-group preservation not independently audited	Precision 71.53%; recall 75.60%; F1 73.51% ^c^
*Condition-specific assessment within a bipolar cohort*				
Temporal multimodal model (2022) [18]	BD corpus; three-state classification	Facial features, audio, text	104 training recordings; result on 60-recording development set; 54-recording test not evaluated	Adaptive late fusion: accuracy 0.9167; UAR 0.8836; not a held-out challenge test result

^*a*^ DNet separately reports a 20-seed AVEC 2014 analysis with different point estimates: MAE 6.1711 (95% CI 6.1490–6.1931) and RMSE 7.7241 (95% CI 7.6880–7.7602). These intervals must not be attached to its headline values. ^*b*^ The fused result is not a Freeform-only result. ^*c*^ The F1 averaging convention was not explicitly resolved; comparison with weighted F1 therefore requires caution. Participant-level performance confidence intervals were not identified for the other tabulated results. The bipolar result is from a development partition and is not directly comparable with the challenge test rows. Its label terminology varies within the primary report; it is reported here only as the source’s three-state task.

**Table 8:** Additional methods and limits of their quantitative interpretation. Values are retained with the evidence available for their evaluation setting. These rows cannot support a direct ranking against Table 7. A dash denotes qualitative coverage without a numerical result included in this synthesis.

Method	Task/Input	Traceable Reported Value	Protocol or Reporting Limitation
LMTformer [61]	AVEC 2013/2014 BDI-II regression; LMVD classification; video	AVEC 2013 MAE/RMSE 6.12/7.75; AVEC 2014 6.05/7.97; LMVD F1 82.74%	Values from the publisher abstract; this evidence alone does not establish partition details or F1 averaging.
LOGLformer [62]	AVEC 2013/2014 scale estimation; facial video	—	Qualitative coverage of local/global facial modelling; no numerical ranking is drawn from this row.
WavFace [63]	DAIC-WOZ screening; audio and facial features	Abstract balanced accuracy 0.81	One-question setting; the cited abstract does not establish sample selection, label rule or participant split.
MMFformer [64]	D-Vlog/LMVD proxy-label classification; audio and video	Reported weighted F1 0.9092/0.9048	Ten-fold evaluation; participant/channel grouping is not established by the evidence used here. D-Vlog uses intermediate-transformer fusion; LMVD uses late-transformer fusion. The reported weighted-recall and accuracy definitions do not support direct equivalence with the earlier benchmark.
Frame-aligned iPPG [57]	54 students, ages 17–23; induced stress versus baseline; pulse features	Approximately 98% accuracy for logistic regression (Sec. IV-C)	Randomised five-fold evaluation; participant grouping not established. Nearby text reports inconsistent point values; no cross-study gain is inferred.
MTASR [59]	UBFC-Phys; experimental stress state/level; video-derived pulse	Accuracy 94.33%/83.83%	Values from the primary abstract; participant partition and uncertainty not specified there. No matched gain over other stress rows is inferred.

## Data Availability

No new data were created or analysed in this review. The datasets and published sources discussed are identified in the article; access is subject to their providers’ conditions.

## Abbreviations

The following abbreviations are used in this manuscript:

AI	Artificial Intelligence
AU	Facial action unit
AUC	Area under the receiver operating characteristic curve
AVEC	Audio/Visual Emotion Challenge
BD	Bipolar disorder
BDI-II	Beck Depression Inventory-II
BVP	Blood volume pulse
CCC	Concordance correlation coefficient
CI	Confidence interval
CNN	Convolutional neural network
CV	Cross-validation
DAIC	Distress Analysis Interview Corpus
DASS-21	Depression Anxiety Stress Scales, 21-item version
ECG	Electrocardiogram
EDA	Electrodermal activity
F1	Harmonic mean of precision and recall
GAD-7	Generalized Anxiety Disorder, 7-item scale
GCN/GNN	Graph convolutional network/graph neural network
HR/HRV	Heart rate/heart rate variability
iPPG	Imaging photoplethysmography
LGBP-TOP	Local Gabor binary patterns from three orthogonal planes
LLM	Large language model
LSTM	Long short-term memory
MAE	Mean absolute error
MiT	Moments in Time
NIR	Near-infrared
NPV/PPV	Negative/positive predictive value
PHQ-8/PHQ-9	Patient Health Questionnaire, 8-/9-item version
PRV	Pulse rate variability
RGB	Red, green and blue
RMSE	Root mean square error
RMSSD	Root mean square of successive interval differences
ROI	Region of interest
rPPG	Remote photoplethysmography
SDNN	Standard deviation of normal-to-normal intervals
SVR	Support vector regression
UAR	Unweighted average recall
ViT	Vision transformer
